# A Dietary Mobile App for Patients Undergoing Hemodialysis: Prospective Pilot Study to Improve Dietary Intakes

**DOI:** 10.2196/17817

**Published:** 2020-07-20

**Authors:** Cosette Fakih El Khoury, Rik Crutzen, Jos M G A Schols, Ruud J G Halfens, Mirey Karavetian

**Affiliations:** 1 Department of Health Services Research Care and Public Health Research Institute, Faculty of Health, Medicine and Life Sciences Maastricht University Maastricht Netherlands; 2 Department of Health Promotion Care and Public Health Research Institute, Faculty of Health, Medicine and Life Sciences Maastricht University Maastricht Netherlands; 3 Department of Family Medicine Care and Public Health Research Institute, Faculty of Health, Medicine and Life Sciences Maastricht University Maastricht Netherlands; 4 Department of Health Sciences Zayed University Dubai United Arab Emirates

**Keywords:** mHealth, dietary app, hemodialysis diet

## Abstract

**Background:**

Mobile technology has an impact on the health care sector, also within dietetics. Mobile health (mHealth) apps may be used for dietary assessment and self-monitoring, allowing for real-time reporting of food intakes. Changing eating behaviors is quite challenging, and patients undergoing hemodialysis, particularly, struggle to meet the target intakes set by dietary guidelines. Usage of mobile apps that are developed in a person-centered approach and in line with recommendations may support both patients and health care practitioners.

**Objective:**

This study is a pilot that aims at estimating the potential efficacy of a dietary intervention using a theory-based, person-centered smartphone app. Results will be used to improve both the app and a planned large-scale trial intended to assess app efficacy thoroughly.

**Methods:**

A prospective pilot study was performed at the hemodialysis unit of Al Qassimi Hospital (The Emirate of Sharjah). All patients that fulfilled the study inclusion criteria were considered eligible to be enrolled in the pilot study. Upon successful installation of the app, users met with a dietitian once a week. Outcomes were measured at baseline (T0) and 2 weeks post app usage (T1). This pilot is reported as per guidelines for nonrandomized pilot and feasibility studies and in line with the CONSORT 2010 checklist for reporting pilot or feasibility trials.

**Results:**

A total of 23 patients completed the pilot intervention. Mean energy intakes increased from 24.4 kcal/kg/day (SD 8.0) to 29.1 kcal/kg/day (SD 7.8) with a medium effect size (*d*=0.6, 95% CI 0.0-1.2). Mean protein intakes increased from 0.9 g/kg/day (SD 0.3) to 1.3 g/kg/day (SD 0.5) with a large effect size (*d*=1.0, 95% CI 0.4-1.6); mean intake of high biological value (%HBV) proteins also increased from 58.6% (SD 10.1) to 70.1% (SD 10.7) with a large effect size (*d*=1.1, 95% CI 0.5-1.7). Dietary intakes of minerals did not change, apart from sodium which decreased from a mean intake of 2218.8 mg/day (SD 631.6) to 1895.3 mg/day (SD 581.0) with a medium effect size (*d*=0.5, 95% CI 0.1-1.1). Mean serum phosphorus, potassium, and albumin levels did not change relevantly. Mean serum iron increased from 7.9 mg/dL (SD 2.8) to 11.5 mg/dL (SD 7.9) postintervention with a medium effect size (*d*=0.6, 95% CI 0.0-1.2).

**Conclusions:**

This pilot study showed that the KELA.AE app has the potential to improve dietary intakes. Processes related to procedure, resources, tools, and app improvement for a future trial were assessed. A more extended intervention using a randomized controlled trial is required to estimate parameters concerning app efficacy accurately.

## Introduction

Mobile technology has the potential to improve health care coverage, especially in low- to middle-income countries where people may be more likely to have access to a smartphone than to basic needs such as safe water and electricity [1]. Self-tracking and wearable technologies have become popular, particularly in the areas of diet and fitness [2]. Previous research on mobile health (mHealth) interventions provides evidence regarding their effectiveness [3-5]. However, their role as educational tools or as supportive tools to standard care remains inconclusive [3,6].

Mobile technology may assist in the introduction of new methods of dietary assessment and self-monitoring, allowing for real-time reporting of food intakes [7]. Self-monitoring is an important factor in successful dietary behavior changes [8]. In a previous study on a weight loss intervention, the more participants recorded food intakes on a mobile app, the more likely they were to lose weight [9]. However, patients may not always be interested and willing to track intakes [10], and commitment to self-monitoring decreases over time, even with the use of mobile technology [11].

Changing eating behaviors is difficult [12], and adherence to guidelines is challenging, especially for patients with chronic conditions such as chronic kidney disease (CKD) [13]. In particular, patients undergoing hemodialysis struggle to meet the target intakes set by dietary guidelines [14]. The diet during hemodialysis is somewhat restrictive, requiring the management of potassium, phosphorus, sodium, and fluids while maintaining adequate protein and energy intakes to prevent malnutrition [15]. In a recent study assessing adherence to diet guidelines among patients undergoing hemodialysis, 77% and 50% of the patients, respectively, consumed less energy and proteins than recommended. These low intakes could be attributed to the restrictive nature of the dialysis diet. In the same study, participants were also found to consume excessive saturated fats and inadequate intakes of fibers and micronutrients [14]. Maintaining adequate intake of minerals such as potassium, phosphorus, and calcium is essential in the prevention of dialysis-related complications such as heart failure, metabolic bone disorders, and mortality [16,17]. Protein-energy malnutrition is also a contributor to complications, and malnutrition among patients undergoing dialysis is a predictor of mortality [18]. Accordingly, it seems that the diet quality of these patients is often poor and that they do not follow dialysis-specific guidelines [14,19].

The main challenge in changing dietary behavior is developing interventions that are comprehensive and sustainable, promoting long-term changes in eating habits and lifestyle [12]. There is a need for nephrologists and dietitians to adopt approaches that strengthen educational and clinical interventions [14]. Possibly, mobile technology could be used to enhance dietetic practice by providing support to patients and dietitians. Thus, adding mobile apps to in-person counseling may provide more accessible and flexible dietetic services at lower costs [20]. However, research on the role of mHealth in dietary behavior in patients undergoing hemodialysis is still scarce [21].

This is a pilot study that aims at estimating the potential of a dietary intervention using a smartphone app in patients undergoing hemodialysis. The results from this pilot study will be used to improve the app itself as well as the study design of the planned randomized controlled trial.

## Methods

### Participants

All patients at the hemodialysis unit of Al Qassimi Hospital (The Emirate of Sharjah) who fulfilled the study inclusion criteria were considered eligible to be enrolled in the pilot study. Inclusion criteria were patients undergoing hemodialysis for at least three months; free of life-threatening conditions; able to read, write, listen, and communicate; owning an Android smartphone; and not having been hospitalized in the past 6 months. All patients approached who accepted to enroll in the study signed written consent forms. A total of 26 participants were enrolled in the pilot, of which 23 downloaded and used the app for 2 weeks. Data were collected between February 2019 and April 2019. Post-hoc calculations of sample size in pilot studies, assuming detection of unanticipated problems with a probability of at least 15% (π=0.15) and a 95% confidence level, resulted in a required sample of 19 patients [22].

### KELA.AE App

**Figure 1:**
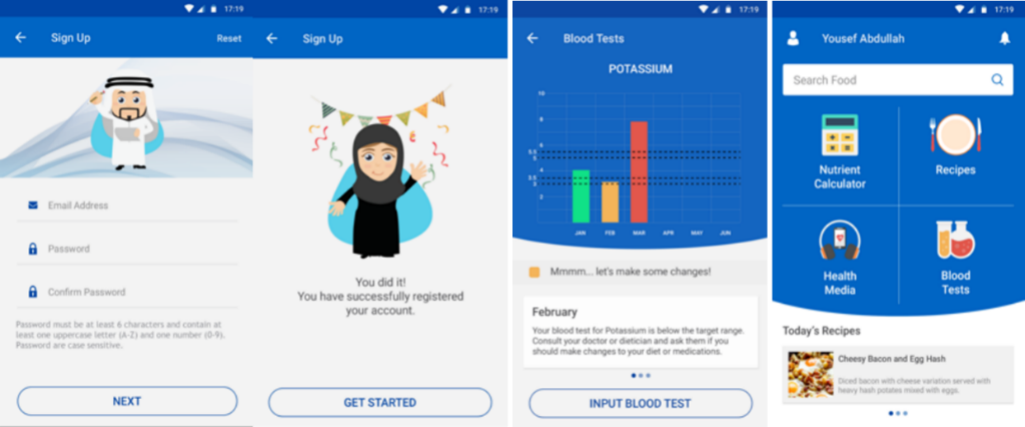
Screenshots of the KELA.AE app used during the pilot study.

KELA.AE (Kidney Education for Lifestyle Application) is an Arabic, culturally specific, educational, and self-monitoring app that was developed in a person-centered, theory-based approach. Educational materials were developed based on the transtheoretical model [23] and constructs of the reasoned action approach [24]. Included educational materials are podcasts, videos, notifications, and recipes. The app also provides self-monitoring features that allow patients to track food intakes and blood parameters. The app was developed following the IDEAS (*i*ntegrate, *de*sign, *a*ssess, and *s*hare) framework [25]; a detailed description of the app development is described elsewhere [10]. Figure 1 depicts screenshots from the app.

### Procedure

This pilot is reported as per guidelines for nonrandomized pilot and feasibility studies [26] and in line with the CONSORT 2010 checklist for reporting a pilot or feasibility trial (items pertinent to randomization were considered not applicable) [27]. Ethical approval was received from the Institutional Review Board of Zayed University, Dubai (Ethical Approval number ZU17_066_F).

Participants who consented to enroll in the pilot study downloaded the app and were provided with usernames that allowed them to sign-in to the app. Upon successful installation of the app, users met with a dietitian once a week (two times in total); the dietitians responded to concerns pertinent to app usage, as well as questions about the renal diet by reinforcing the critical messages relayed by the educational materials. The dietitians also collected the data from patients before and after app usage. All outcomes were measured before (T0) and after 2 weeks (T1) of app usage.

### Dietary Intakes

Face-to-face 24-hour recalls were collected [28] at T0 and T1 for all participants; nutrient compositions were derived for energy, carbohydrates, proteins, high biological value (%HBV) proteins, total fat, potassium, phosphorus, and sodium. Intakes were then compared with dietary guidelines for patients undergoing hemodialysis [15,29]. Protein targets were considered as 1.2 g/kg or more with 50% or higher HBV protein; energy as 30-35 kcal/kg; phosphorus as 1000 mg/day for participants with serum phosphorus below 5.5 mg/day and 12 mg/g of protein intake for participants with serum phosphorus below 5.5 mg/dL. Sodium and potassium targets were considered less than 2400 mg/day [15,29]. Standard body weight from the National Health and Nutrition Examination Study was used for calculations; however, adjusted edema-free body weight was used for calculating nutrient needs for individuals with less than 95% or greater than 115% of standard body weight [29]. Textbox 1 illustrates the dietary guidelines that were used as targets and that are also included in the adherence index.

**Dietary guidelines for patients undergoing hemodialysis used for adherence index [15,29]. HBV: high biological value, PTH: parathyroid hormone, aBW_ef_: adjusted edema-free body weight, SBW: standard body weight, BW_ef_: edema-free body weight.**

1. Protein≥1.2 g/kg of body weight, ≥50% HBV protein
2. Energy for those <60 years of age: kilogram (kg) of body weight × 35 kcal; >60 years of age: kilogram (kg) of bodyweight × 30 kcal/kg to 35 kcal/kg
3. Sodium less than 2.4 g/day
4. Potassium less than 2.4 g/day
5. Phosphorus 800 mg/day to 1000 mg/day or 10-12 mg phosphorus/g of protein when serum phosphorus >5.5 mg/dL or intact PTH is elevated
6. aBW_ef_ is recommended for calculating nutrient needs for individuals with <95% or >115% of SBW using the formula aBW_ef_ (kg) = BW_ef_+ [(SBW – BW_ef_) × 0.25]. SBW from the National Health and Nutrition Examination Study is used otherwise

### Biochemical Parameters

Serum phosphorus, potassium, and iron were retrieved from the patients’ medical records. These biochemical parameters were measured as part of the routine tests performed in the hemodialysis unit. All tests were conducted postdialysis (in sessions). Comparative standards for serum potassium were considered between 3.5 and 5.5 mEq/L; and between 3.5 and 5.5 mg/dL for phosphorus [15,29].

### Statistical Analysis

The Shapiro–Wilk normality test was performed to ensure that data are normally distributed. Cohen *d* effect sizes and confidence intervals (95%) were derived from means and pooled standard deviations. The effect size was considered small at 0.2, medium at 0.5, and large at 0.8 and above [30]. Effect sizes were calculated to understand the magnitude of the reported effects along with the probability by means of *P* values [31]. Paired *t* tests were performed to compare the mean scores before and after the intervention. Two-tailed *P* values are reported. Frequencies and percentages were used to describe categorical variables, whereas means and standard deviations were used for continuous variables. Statistical software IBM SPSS Statistics 21 Data Editor was used to perform all statistical analyses.

## Results

### Participant Eligibility and Baseline Characteristics

**Figure 2:**
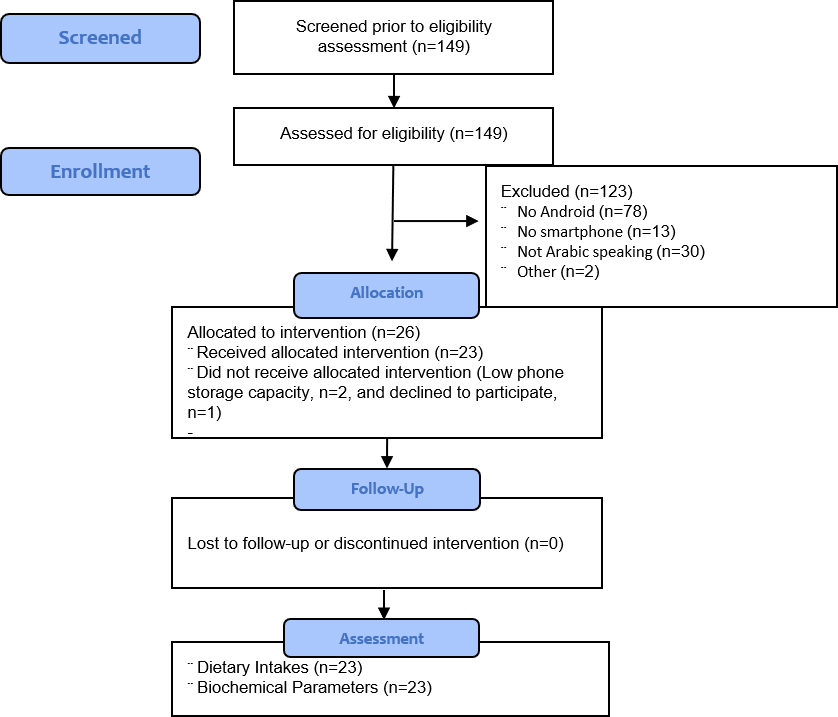
CONSORT study flow diagram.

Of the 149 patients at the hemodialysis unit, 26 were eligible, of which 23 downloaded the app and completed the study. Participants that were not eligible were mainly those who did not own a smartphone or who owned a smartphone with an iOS operating system; 2 participants did not download the app due to limitations in phone storage, and 1 was not interested in downloading the app. Figure 2 depicts the CONSORT flow diagram. The sample’s mean age (years) was 48.5 (SD 13.7), and mean BMI (kg/m^2^) was 31.9 (SD 7.9); participants had been on dialysis for a mean of 29.7 (SD 37.3) months. More than half of the participants were males (n=14), and more than half suffered from hypertension or diabetes or both. Demographic data are detailed in Table 1.

**Table 1:** Demographic and baseline characteristics of the sample (N=23).

Characteristics			Value
Age, mean (SD)			48.5 (13.7)
BMI, mean (SD)			31.9 (7.9)
Months on dialysis, mean (SD)			29.7 (37.3)
**Gender, n (%)**			
	Male	14 (61)	
Smokers, n (%)		6 (26)	
**Comorbidities, n (%)**			
	Hypertension	16 (70)	
	Diabetes	11 (48)	
	Dyslipidemia	2 (9)	
	Cancer	1 (4)	
	Liver disease	1 (4)	

### Anthropometry and Dietary Intakes

There was no change in weight and BMI postintervention; however, mean dietary intakes changed mainly for energy and macronutrients. Mean energy intakes increased from 24.4 kcal/kg/day (SD 8.0) to 29.1 kcal/kg/day (SD 7.8) with a medium effect size (*d*=0.6, 95% CI 0.0-1.2). Mean protein intakes increased from 0.9 g/kg/day (SD 0.3) to 1.3 g/kg/day (SD 0.5) with a large effect size (*d*=1.0, 95% CI 0.4-1.6); mean intake of HBV protein (%) also increased from 58.6% (SD 10.1) to 70.1% (SD 10.7) with a large effect size (*d=*1.1, 95% CI 0.5-1.7). Total fat intakes increased from baseline with medium effect size (*d=*0.5, 95% CI 0.0-1.1). Dietary intakes of minerals did not change, apart from sodium which decreased from a mean intake of 2218.8 mg/day (SD 631.6) to 1895.3 mg/day (SD 581.0) with a medium effect size (*d=*0.5, 95% CI 0.1-1.1).

### Biochemical Parameters

Serum phosphorus, potassium, and albumin did not change relevantly. Mean serum iron increased from 7.9 mg/dL (SD 2.8) to 11.5 mg/dL (SD 7.9) postintervention with a medium effect size (*d=*0.6, 95% CI 0.0-1.2). Table 2 details the results of the anthropometry, dietary intakes, and blood parameters.

**Table 2:** Dietary intakes and laboratory data at baseline and postintervention.

Dietary intakes and laboratory data	Baseline (T0), mean (SD)	Postintervention (T1), mean (SD)	Cohen *d* (95% CI)	*P* value
Weight (kg)	85.5 (23.1)	84.1 (24.1)	0.1 (–0.5 to 0.6)	.37
BMI (kg/m^2^)	31.9 (7.9)	31.3 (8.5)	0.1 (–0.5 to 0.6)	.39
Energy intake (kcal/day)	1918.3 (570.4)	2206.2 (378.2)	0.6 (0.0 to 1.2)	.003*
Energy intake (kcal/kg/day)	24.4 (8.0)	29.1 (7.8)	0.6 (0.0 to 1.2)	.002*
Dietary protein (g/day)	71.1 (26.4)	103.8 (37.8)	1.0 (0.4 to 1.6)	<.001*
Dietary protein (g/kg/day)	0.9 (0.3)	1.3 (0.5)	1.1 (0.4 to 1.7)	<.001*
HBV protein^a^ (%)	58.6 (10.1)	70.1 (10.7)	1.1 (0.5 to 1.7)	<.001*
Total dietary CHO^b^ (g/day)	224.7 (88.0)	215.2 (40.7)	0.1 (–0.4 to 0.7)	.59
Total dietary fat (g/day)	87.3 (30.1)	103.0 (26.8)	0.5 (0.0 to 1.1)	.02*
Dietary potassium (mg/day)	1831.2 (728.4)	2046.1 (555.1)	0.3 (0.2 to 0.9)	.19
Dietary phosphorus (mg/day)	1152.5 (489.7)	1343.1 (398.0)	0.4 (0.2 to 1.0)	.09
Dietary sodium (mg/day)	2218.8 (631.6)	1895.3 (581.0)	0.5 (0.1 to 1.1)	.03*
Serum potassium (mg/dL)	4.7 (0.7)	4.7 (0.7)	0.0 (–0.6 to 0.6)	.92
Serum phosphorus (mg/dL)	5.2 (1.5)	5.5 (2.2)	0.15 (–0.4 to 0.7)	.60
Serum iron (mg/dL)	7.9 (2.8)	11.5 (7.9)	0.6 (0.0 to 1.2)	.03*
Serum aluminum (g/dL)	3.0 (0.4)	3.2 (0.8)	0.3 (–0.3 to 0.9)	.37

*P<.05.

^a^HBV: high biological value.

^b^CHO: carbohydrates.

### Adherence to Dietary Guidelines in Hemodialysis

**Figure 3:**
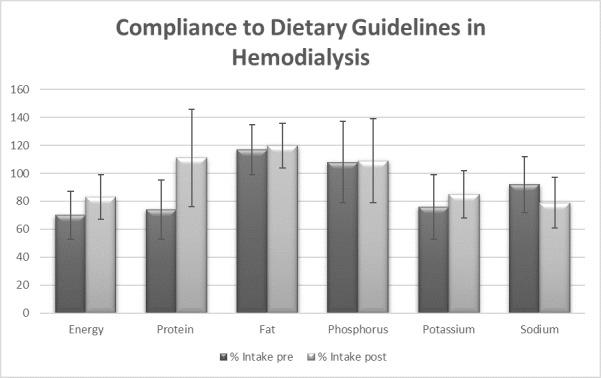
Percent (%) adherence to dietary intakes pre- and post-intervention. Values are presented as mean (SD).

Adherence to dietary guidelines in hemodialysis improved for energy intakes, protein intakes, and %HBV proteins with medium to large sizes, respectively (*d=*0.4, 95% CI 0.2-1.0 for energy; *d=*0.9, CI 0.3, 1.5 for proteins; *d=*1.1, 95% CI 0.5-1.7 for %HBV proteins). Adherence to fat, potassium, and phosphorus intakes did not change, whereas adherence to sodium further dropped to achieve intakes below 2400 mg/day with a medium effect size (*d=*0.5, CI 0.1-1.1). The number of patients adhering to dietary guidelines increased for energy, proteins, and sodium, whereas a larger number of patients became nonadherent to fat and phosphorus intakes. There was no relevant change in the number of patients concerning adherence to potassium intakes. Table 3 describes the difference between the recommended intakes and dietary intakes before and after the intervention. Figure 3 details the results of percent compliance to dietary recommendations.

**Table 3:** Adherence to dietary intakes reported as the difference between intake and recommendation at baseline and postintervention.

Dietary intakes	Baseline, mean (SD)	Postintervention, mean (SD)	Cohen *d* (95% CI)	*P* value
Energy (kcal/day)	–903.1 (705.4)	–581.3 (779.8)	0.4 (0.2 to 1.0)	.004*
Dietary protein (g/day)	–27.3 (26.6)	+6.5 (43.9)	0.9 (0.3 to 1.5)	<.001*
HBV proteins^a^ (%)	58.6 (10.1)	70.1 (10.7)	1.1 (0.5 to 1.7)	<.001*
Total dietary fat (% energy)	41.0 (7.6)	41.9 (7.3)	0.1 (–0.7 to 0.4)	.66
Dietary potassium (mg/day)	–568.1 (728.4)	–353.9 (555.1)	0.3 (–0.9 to 0.2)	.19
Dietary phosphorus (mg/day)	+84.7 (445.8)	250.3 (369.9)	0.4 (–0.9 to 0.2)	.14
Dietary sodium (mg/day)	–181.1 (631.6)	–504.6 (581.0)	0.5 (0.1 to 1.1)	.03*

^a^HBV: high biological value.

### User Acceptability and App Usability

A user acceptability tool was used during this pilot; however, all participants responded to all questions with a *strongly agree* option based on a Likert scale. Accordingly, the results were not considered useful to understand better the acceptability and, therefore, have not been reported. Assessment of acceptability will be reassessed in the future trial. The validated end-user version of the Mobile App Rating Scale (uMARS) [32] will be used in the trial. Participants may not have well understood that the questionnaire aims to improve the app; thus, qualitative data will also be collected to ask specific questions about the KELA.AE usability and acceptability by referring to each feature in the app individually. Qualitative data will also address the culturally specific features of the app such as language and recipes.

Field notes revealed that all 23 participants attended the weekly sessions with the dietitians. Usage data available from analytics were only able to provide the last date of access for each participant, and all 23 had accessed the app in the last week of the trial. No data on usage frequency could be retrieved due to a lack of in-app analytics.

## Discussion

### Principal Results

#### Potential Efficacy

In this study, short-term app usage had a potential impact on energy and protein intakes among patients undergoing hemodialysis. In comparison to the target dietary guidelines for hemodialysis, patients started with a baseline intake of energy and proteins below recommendations. As an outcome of the intervention, mean energy intakes reached 29.1 kcal/kg/day (SD 7.8) as compared with the recommended 30-35 kcal/kg/day. In addition, protein intakes were also considerably low as compared with guidelines (mean consumption at baseline of 0.9 g/kg/day [SD 0.3]) and improved to become in line with the recommended 1.2 g/kg or more of body weight and 50% or higher HBV protein [15,29].

No changes were observed in intakes of minerals; nonetheless, baseline intakes of potassium and sodium were already within the recommendations of less than 2400 mg/day. Mean sodium intakes further dropped to 1895.3 mg/day (SD 581.0). Fat and phosphorus intakes, however, were already elevated at baselines and remained above recommendations after the intervention.

No changes were observed in the serum laboratory parameters for potassium and phosphorus; levels remained within recommendation at baseline and after the intervention. The duration of the intervention is not long enough to detect relevant changes in serum values; accordingly, the upcoming trial is expected to capture the effect of app usage on laboratory parameters better. Mean serum iron increased with a medium effect size from 7.9 mg/dL (SD 2.8) to 11.5 mg/dL postintervention. The latter may be explained by the increase in the dietary protein of HBV, which mainly consists of animal protein sources.

#### Study and App Feasibility

Research methodology, resources, and tools used in this pilot were assessed to refine and modify the planned trial and improve the app. The feasibility of the processes related to recruitment, retention, and refusal seemed adequate; there were no patients that withdrew from the study; and all eligible patients agreed to participate. However, the number of eligible patients were about 17.4% (26/149) out of all patients undergoing dialysis at the unit. It would mean that to achieve an appropriate sample size for the planned randomized trial, many dialysis units will need to be recruited. Thus, the number of dietitians needed to be involved in the future study will need adequate resources to meet patients weekly, as it was the case in this study. Accordingly, an assessment of resource allocation should be performed to consider app improvement and development on iOS mobile operating system to increase eligible patients within the same dialysis unit.

Digital interventions may be used as tools to support a reciprocal relationship between patients and health care practitioners and enhance patient-centered care [33]. Accordingly, dietitians would be expected to manage and recommend the usage of KELA.AE app in real practice settings. Thus, the future trial will include dietitians following up with patients to simulate app integration in real practice.

In-app analytics are also essential for the future trial to assess the effect of app usage on outcomes. In-app analytics should track usage frequency as well as the usage of each feature separately. The results would help to better understand if educational or self-monitoring features of the app are preferred by the user.

The future trial should further analyze the fatty acid profile (saturated, monounsaturated, and polyunsaturated) on top of total fats to assess diet quality from an atherogenic point of view. In addition, further assessment of the nutritional status of patients undergoing dialysis should be performed to identify patients with malnourishment that may need additional support from the research dietitians.

The KELA.AE app will be re-evaluated to include educational materials addressing diet quality pertinent to dietary fatty acid profiles to promote dietary intakes in line with the KDOQI Clinical Practice Guidelines for the Management of Dyslipidemias in Patients With Kidney Disease [34]. This is an update to be considered in the next version of the app should dietary fat intakes not be in line with guidelines.

This pilot study has demonstrated that it is feasible to integrate a dietary app into dietetic practice, allowing it to be a tool in addition to regular meetings with dietitians. The study has also identified how to adjust the design, procedures, data collection tools, and outcome measurements for a future trial. Based on the information collected, we feel ready to proceed to a larger randomized controlled trial.

### Comparison With Prior Work

Energy and protein recommendations in hemodialysis are higher than the recommendations for healthy individuals. Whereas in the general population, intakes of 0.8 g/kg are adequate to maintain nitrogen balance [35], such intakes are inadequate in hemodialysis [36]. Hemodialysis sessions cause losses in proteins and amino acids that need to be replenished by the diet [36]. Protein-energy wasting is dangerous and has been correlated with mortality, adverse clinical outcomes [37], and poor quality of life [18]. Both Inadequate dietary proteins and energy intakes are criteria used in the diagnosis of protein-energy wasting in patients undergoing hemodialysis [38]. Similar to the findings in this pilot, many studies have identified inadequate energy and protein intakes among patients undergoing dialysis [14,39,40]. This pilot intervention seems to show potential in the improvement of energy and protein intakes among this population.

However, multiple dietary components need to be adjusted to improve the clinical outcomes of these patients, and the latter includes management of potassium and phosphorus, along with a diet quality that is cardioprotective [15]. In the sample studied in this pilot, both serum potassium levels and potassium intakes were within targets before and after the intervention. This was also observed by others [14,19], whereby similar to our findings, patients undergoing hemodialysis tend to be adherent to potassium intakes and laboratory targets. However, serum phosphorus and phosphorus intakes were borderline high both before and after the intervention. This is also a common finding in this population, in which the discrepancies between the phosphorus and protein recommendations [15] make it challenging to achieve the required targets. Accordingly, it seems that further assessment of the factors that may influence phosphorus management, such as food sources of phosphorus and compliance to phosphate binders, should be explored. In addition, it is expected that a longer intervention that includes education and follow-up with dietitians may lead to better outcomes.

Total fat intakes were also found to be elevated by our pilot study, and their intakes remained elevated after the intervention as well. Others have previously explored the diet quality of patients undergoing hemodialysis as compared with the recommendations of the American Heart Association and KDOQI Clinical Practice Guidelines for Management of Dyslipidemias in Patients With Kidney Disease [34], and found the current diet intakes to be proatherogenic in nature [14]. Given that protein intakes increased and that their sources are mainly from animal proteins (an increase in %HBV proteins were also observed), it can be predicted that saturated fat intakes also increased as a result of these changes.

There are only few available apps that target patients with CKD specifically; however, most of them are available on app stores, and information on their efficacy and usability are rather scarce [41]. A recent content analysis of mobile apps for CKD revealed that available apps fail to provide the continuity of patient-centered care that is needed to support patients with CKD [42].

### Limitations

Given the pilot nature of the study, some limitations may lead to a bias in the interpretation of the results and their generalizability. The duration of this study was short; therefore, its effects are only focused on dietary intakes and laboratory parameters. Behavioral interventions need to be of longer duration and should be comprehensive to change dietary intakes [43]. The results of this pilot will instead be used to improve the app and modify the study procedures in the future trial.

In addition, the results identified a possible improvement in nutritional status and a potentially proatherogenic diet quality among the study’s sample. Thus, the future trial should assess the prevalence of patients with malnourishment along with the fatty acid profile of the diet in line with the KDOQI Clinical Practice Guidelines for Management of Dyslipidemias in Patients With Kidney Disease [34].

The intervention included face-to-face sessions with dietitians that may have influenced the outcomes. The reinforcement of the dietitians and their availability may have impacted the results similarly or more than the app itself. Accordingly, the future trial should include a control group whereby dietitians see patients with the same frequency but without app usage.

Given that app analytics were not available, we were unable to track how many times the users accessed the app. The only available data are the last access for each user, and all users had accessed the app during the last week.

### Conclusions

This pilot study showed that KELA.AE app has the potential to improve dietary intakes. Processes related to the procedures, resources, tools, and app improvement for a future trial were assessed. A more extended intervention using a randomized controlled trial is required to estimate parameters concerning app efficacy accurately.
